# Different Ultrasound Shear Wave Elastography Techniques as Novel Imaging-Based Approaches for Quantitative Evaluation of Hepatic Steatosis—Preliminary Findings

**DOI:** 10.3390/tomography9020054

**Published:** 2023-03-16

**Authors:** Natascha Platz Batista da Silva, Gregor Scharf, Lukas Lürken, Niklas Verloh, Stephan Schleder, Christian Stroszczynski, Ernst Michael Jung, Michael Haimerl

**Affiliations:** 1 Department of Radiology, University Hospital Regensburg, Franz-Josef-Strauß-Allee 11, 93053 Regensburg, Germany; 2 Department of Diagnostic and Interventional Radiology, Medical Center University of Freiburg, Hugstetter Straße 55, 79106 Freiburg im Breisgau, Germany; 3 Department of Diagnostic and Interventional Radiology, Merciful Brothers Hospital St. Elisabeth, 94315 Straubing, Germany; 4 Department of Diagnostic and Interventional Radiology, Hospital Wuerzburg Mitte, 97074 Wuerzburg, Germany

**Keywords:** shear-wave elastography, shear-wave dispersion, attenuation imaging, hepatic steatosis, liver MRI, PDFF

## Abstract

Background: Modern ultrasound (US) shear-wave dispersion (SWD) and attenuation imaging (ATI) can be used to quantify changes in the viscosity and signal attenuation of the liver parenchyma, which are altered in hepatic steatosis. We aimed to evaluate modern shear-wave elastography (SWE), SWD and ATI for the assessment of hepatic steatosis. Methods: We retrospectively analyzed the US data of 15 patients who underwent liver USs and MRIs for the evaluation of parenchymal disease/liver lesions. The USs were performed using a multifrequency convex probe (1–8 MHz). The quantitative US measurements for the SWE (m/s/kPa), the SWD (kPa-m/s/kHz) and the ATI (dB/cm/MHz) were acquired after the mean value of five regions of interest (ROIs) was calculated. The liver MRI (3T) quantification of hepatic steatosis was performed by acquiring proton density fat fraction (PDFF) mapping sequences and placing five ROIs in artifact-free areas of the PDFF scan, measuring the fat-signal fraction. We correlated the SWE, SWD and ATI measurements to the PDFF results. Results: Three patients showed mild steatosis, one showed moderate steatosis and eleven showed no steatosis in the PDFF sequences. The calculated SWE cut-off (2.5 m/s, 20.4 kPa) value identified 3/4 of patients correctly (AUC = 0.73, *p* > 0.05). The SWD cut-off of 18.5 m/s/kHz, which had a significant correlation (r = 0.55, *p* = 0.034) with the PDFF results (AUC = 0.73), identified four patients correctly (*p* < 0.001). The ideal ATI (AUC = 0.53 (*p* < 0.05)) cut-off was 0.59 dB/cm/MHz, which showed a significantly good correlation with the PDFF results (*p* = 0.024). Conclusion: Hepatic steatosis can be accurately detected using all the US-elastography techniques applied in this study, although the SWD and the SWE showed to be more sensitive than the PDFF.

## 1. Introduction

In recent years, different ultrasound (US) shear wave elastography (SWE) techniques have been developed, and new diagnostic approaches can now be used in the non-invasive diagnosis of liver diseases [1,2,3]. Through the application of repetitive high frequency acoustic radiation force impulses into the liver tissue, a qualitative, color-coded elastogram and quantitative measurements of the shear-wave speed (in m/s) or of the stiffness (in kPa) of the elasticity changes in the liver tissue can be obtained for diagnostic and monitoring purposes [1,2]. This enables both the qualitative and quantitative assessments of parenchymal liver diseases, such as hepatic steatosis, inflammatory liver diseases, liver fibrosis and cirrhosis [4,5,6,7].

According to the international European Federation of Societies for Ultrasound in Medicine and Biology (EFSUMB)’s guidelines and recommendations on ultrasound elastography, there are several indications for the hepatic application of US elastography: the detection, staging and monitoring of chronic hepatitis B and C; autoimmune hepatitis; non-alcoholic fatty liver disease (NAFLD and NASH); and alcoholic liver disease (ALD), including cirrhosis and cholestatic liver disease leading to fibrosis [1,2,8]. Transient Elastography (TE) or point SWE (pSWE) techniques are the most commonly recommended techniques rather than 2D SWE, although multiple author groups previously reported a high correlation between 2D SWE measurements and histologically proven hepatitis or hepatic fibrosis/cirrhosis [5,6,7,9,10].

The detection of hepatic steatosis as seen in fatty liver disease can be difficult when a conventional B-mode US is used alone, as a subjective signal-attenuation change compared to renal parenchyma is the only parameter that can be documented [11]. The use of SWE techniques to quantify liver steatosis, which is caused by lipid accumulation in hepatocytes (>5%), seems promising in terms of detection and staging [12,13]. However, conventional SWE techniques seem less able to adequately monitor and detect the slight signal attenuations and changes in the liver parenchyma’s viscosity observed in early hepatic steatosis [5,13,14,15].

Modern shear-wave elastography applications such as shear-wave dispersion (SWD) and attenuation imaging (ATI) can be used to visualize and quantify changes in the viscosity and the signal attenuation of the liver parenchyma, which are known to increase in hepatic steatosis because of the accumulation of lipids inside the cells and the chronic inflammation of hepatocytes [16,17]. SWD quantifies changes in the viscoelasticity of the liver parenchyma [17,18] and ATI enables a real-time visualization of signal attenuation in hepatic steatosis, which can be quantified using a calculated attenuation coefficient [19,20,21,22]. Some studies have already proven the high efficiency of SWE, SWD and ATI in the detection and scoring of hepatic steatoses compared with the results of biopsies or magnetic-resonance-imaging (MRI) techniques [12,14,15,16,17,23]. However, only a few overview studies comparing all three techniques—SWE, SWD and ATI—in correlation with non-invasive proton density fat fracture (PDFF) (which is reported to be an acceptable gold standard for fat quantification in the liver [19,24]) described findings in patients with and without NAFLD, and the precise differentiation parameters were not mentioned in these studies [25]. Consequently, the aim of this study was to evaluate the efficiency of transabdominal USs using qualitative and quantitative SWE, SWD and ATI parameters for the detection of fatty liver disease in relation to non-invasive MR-tomographic PDFF using fat-signal-fraction measurements as a gold standard. Therefore, the first cut-off values for all three techniques for the detection of hepatic steatosis should be suggested.

## 2. Materials and Methods

### 2.1. Study Design

This retrospective proof-of-principle study was conducted in compliance with the Standards for Reporting of Diagnostic Accuracy (STARD) guidelines and the rules of the Declaration of Helsinki. The study was approved by the Institutional Review Board of the University of Regensburg (21-2394-104). Written informed consent for MRI examinations was obtained in each case.

We performed a retrospective analysis of the digitally stored US, MRI and clinical data of 15 consecutive patients who underwent a transabdominal US—including SWE, SWD and ATI examinations—due to the suspicion of fatty liver disease during a one-month time period (11–12/2020). Inclusion criteria were the patient’s age (>18), with no present history of diffuse hepatic tumor disease and no contraindications to undergo an MRI examination of the liver. Eleven male and four female patients underwent an MRI scan of the liver, including PDFF sequences in the axial view in addition to the US examination. The US findings were correlated with the MRI’s fat signal fraction measurement results, which is the current gold standard, to confirm the diagnosis and stage (mild, moderate or severe) of the hepatic steatosis.

### 2.2. US Examinations

All US examinations were performed by experienced examiners using a modern US machine (Aplio i800, Canon Medical Systems, Otawara, Japan) with each patient being placed in the supine position with an elevated right arm to obtain an intercostal view of the liver. A multifrequency convex probe of 1–8 MHz was used for a B-mode US of the liver and for elastography measurements. Images and cine loops from the B-mode, SWE, SWD and ATI techniques were stored digitally on the US machine and in the local PACS. During the qualitative and quantitative analyses, measurements were displayed and documented in tabular form as well as in a color-coded chart form, which classified the acquired measurements as “normal” (green), “mild” (orange), “significant” (yellow) or “severe” (red). Standardized intercostal and subcostal views for the left and right liver lobes were performed in each case.

### 2.3. Shear-Wave Elastography

**Figure 1:**
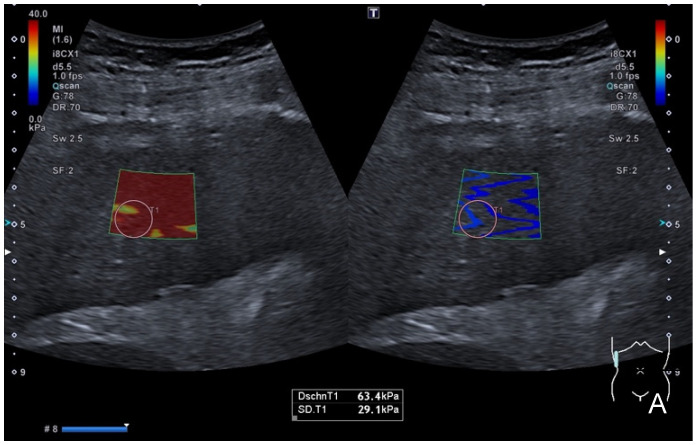

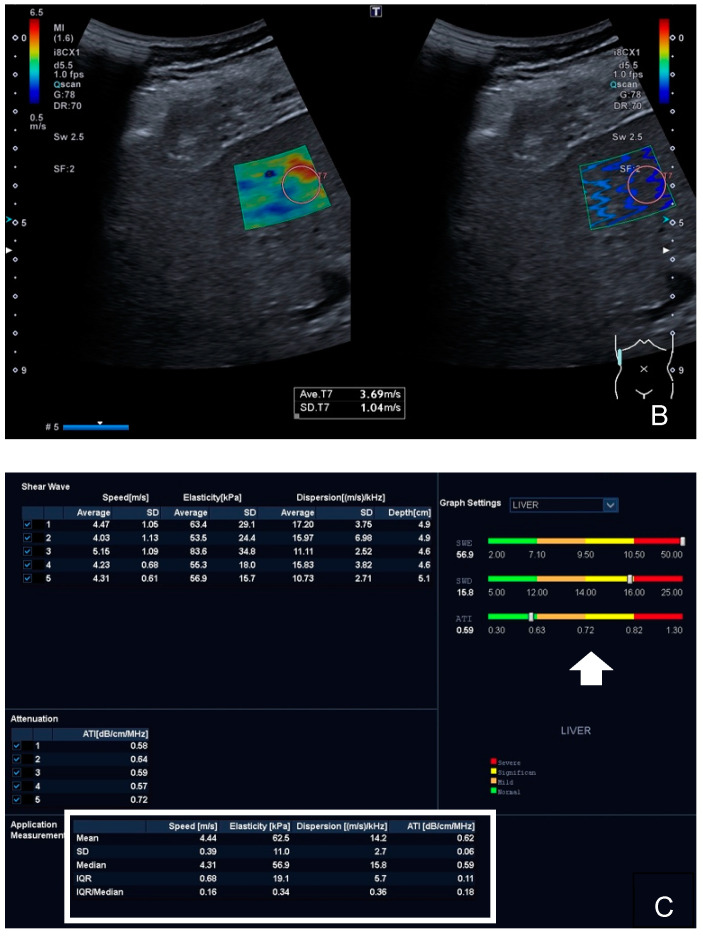
Intercostal view in an SWE application (left in (**A**,**B**)) with corresponding shear-wave dispersion (right in (**A**,**B**)) in a patient with severe liver fibrosis/cirrhosis. (**A**) displays a color-coded elastogram representing the elevated stiffness (red colorization of the sample window), which was confirmed in quantification measurements after placing a sample ROI inside the SWE box which presented stiffness values of 63.4 kPa and an SWD of 14.2 m/s/kHz (**C**). (**B**) shows the corresponding SWS mapping of the same patient with an inhomogenously colored elastogram, representing elevated shear-wave speed values in this case of advanced cirrhosis, which was confirmed in quantitative analysis (SWS = 3.69 m/s). The validity of acquired measurements was confirmed using the dispersion slope form as a quality indicator. (**C**) provides corresponding quantitative “liver analysis” results: mean and median values (±sd) of the SWE, the SWD and the ATI were calculated (white box), and the classification of individual findings was performed according to the color-coded scale (white arrow). This patient’s measurements were classified as “severe” considering the SWE, “significant” considering the SWD and “normal” considering the ATI analysis, suggesting the presence of severe fibrosis or cirrhosis but only little inflammation or steatosis.

After entering the SWE mode, a sample box was placed at an adequate depth, and the SWE’s images and loops were acquired using the standardized breath-hold technique. The homogeneity of the color-coding inside the sample window, as well as a separately provided homogenous wave-propagation image, served as indicators of the quality of the SWE measurements. If no homogenous image could be obtained, the position was altered and the procedure was repeated. In SWE, repetitive acoustic radiation force impulses (ARFIs) are applied in cycles, inducing shear waves in the targeted tissue. Shear waves can be visualized in color-coded maps and their speed can be measured, which correlates with the tissue’s stiffness [1]. To obtain reproducible and quantifiable measurements, a total of five regions of interest (ROIs) sized 10 mm × 10 mm were placed in each case inside the color-coded SWE map in equivalent positions, and the shear-wave speed (SWS) in m/s, and the stiffness in kPa were measured. Mean values (±sd) for SWS and stiffness were calculated from 5 to 6 single measurements. A cut-off value for the detection of liver steatosis using SWE was calculated and applied. The SWE measurements were correlated to the PDFF’s fat-signal-fraction measurements. An example of the application of SWE is provided in Figure 1.

### 2.4. Shear-Wave Dispersion

In addition to the SWE measurements, the shear-wave dispersion of the targeted liver tissue was measured in each patient in (m/s)/kHz. As shear-wave dispersion not only depends on the liver’s elasticity but also on its viscosity, viscoelasticity can be measured as a function of SWS and the corresponding local shear-wave frequency [18]. Automatically, an average value was calculated and displayed (±sd) after a corresponding sample box was placed, which simultaneously color-coded the SWD slope (see Figure 1). Five to six SWD cycles which supplied corresponding measurements were acquired, each using the breath-hold technique, meaning a mean SWD value was calculated for each patient. The SWD measurements were correlated with the PDFF results to assess this technique’s accuracy in the detection of fatty liver.

### 2.5. Attenuation Imaging Technique

**Figure 2:**
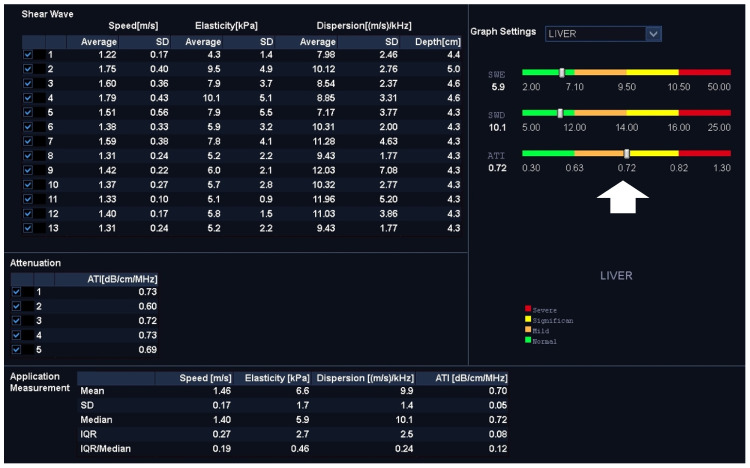
Qualitative and quantitative ATI evaluations after acquisition of 5 ATI cycles (mean 0.7 dB/cm/MHz). The automated, integrated color scale (white thickened arrow) for classification of the patient’s values suggested “mild to significant” changes in ATI analysis, although SWE and SWD measurements were considered “normal”. These findings suggest the presence of significant steatosis with lobular inflammation, but without relevant fibrosis.

ATI was also performed using the breath-hold technique after the acquisition of the SWE and the SWD using the same convex US probe. The ATI technique is based on an automatic subtraction of system-dependent parameters—i.e., internal gain control, transducer factor, beam profile and further artifacts—leading to the display of an adjusted and colored intensity image inside the sample box. The obtained image color-codes the signal attenuation inside the sample box and enables the calculation of an attenuation coefficient [15,21]. This coefficient represents the quantitative, depth-dependent intensity attenuation expressed in dB/cm/MHz. The higher the coefficient, the more attenuation is generated by the examined liver tissue, suggesting the presence of higher levels of steatosis (see Figure 2). Five to six measurements were obtained in each case, and a mean coefficient (±sd) was calculated for each participant. The ATI measurements were correlated with the PDFF results, which was used as a gold standard, to evaluate the ATI’s efficiency in the steatosis assessment.

### 2.6. MRI of the Liver Using Proton Density Fat Fraction (PDFF) Sequences

**Figure 3:**
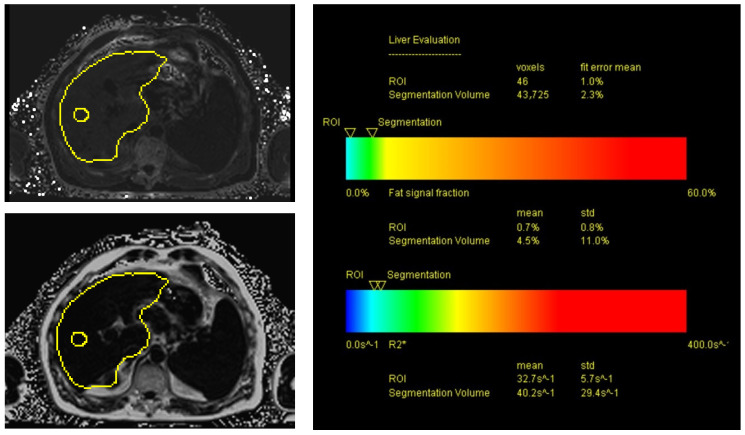
MRI PDFF sequences in axial view of a patient with known liver cirrhosis. The fat signal fraction inside the positioned ROI (see yellow ROI in left images) showed mean values <5%, consistent with no evidence of hepatic steatosis.

**Figure 4:**
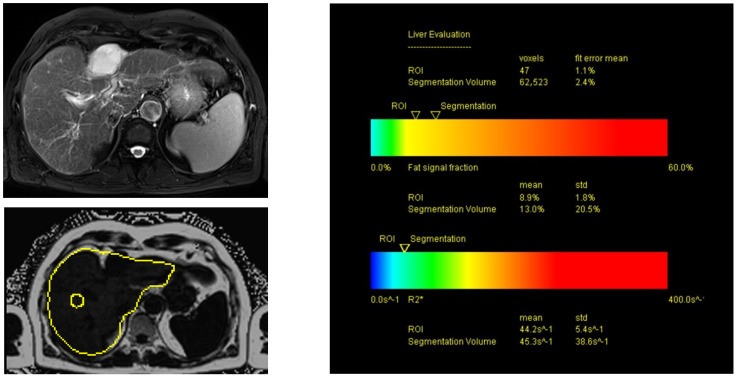
MRI T2-weighted (left upper image, T2 BLADE (fs)) and PDFF mapping (left lower image) in axial view in a patient with hepatic steatosis and initial fibrosis. The fat signal fraction inside the manually placed ROI (yellow circle in left lower image) showed a mean of 8.9%, consistent with mild to moderate hepatic steatosis.

As mentioned above, all the US results were correlated with the PDFF’s fat-signal-fraction measurements after the liver MRIs were carried out. All the MRI examinations were performed on a 3T scanner (MAGNETOM Skyra, Siemens Healthcare) using an 18-channel body coil and elements of a 32-channel spine coil for signal reception. Our standard liver protocol included T2-weighted Turbo Spin Echo (TSE) sequences with and without spectral fat saturation (fs) as well as diffusion-weighted imaging. Before contrast application, a 3D Volumetric Interpolated Breathhold Examination (VIBE) fs sequence was acquired, which was repeated during the arterial, portal venous, late venous and hepatobiliary phase 20 min after the injection of Gd-EOB-DTPA (Primovist^®^; dosage: 0.1 mL/kg bodyweight). Finally, PDFF sequences were acquired with a 6-echo prototype 3D VIBE sequence using a 7-fat-peak model and a multistep adaptive fitting approach (TR = 9.2 ms, TE = from 1.23 to 7.38 ms with ΔTE = 1.23 ms, flip angle = 4°), a measured voxel size of 2.9 mm × 2.6 mm × 6.4 mm (interpolated to 2.6 mm × 2.6 mm × 4.0 mm) and CAIPIRINHA with acceleration factors of “2” in both the phase- and slice-encoding directions. The acquisition time was 15 s. An ROI was positioned manually in an artifact-free area for the inline evaluation of PDFF, measuring the fat signal fraction (in %). Additionally, the fat signal fraction was displayed in a color-coded bar encoding fat fractions of 0–60% in blue, green, yellow, orange and red color shades (Figure 3 and Figure 4).

### 2.7. Statistical Analysis

Statistical analysis was performed using a *t*-test for paired samples; receiver operating characteristic (ROC) curves in combination with the Youden index; and an area under the ROC (AUROC) curve analysis to assess the methods’ accuracy levels. The univariate Pearson’s r coefficient was also used to detect any significant correlations between the SWE and PDFF, SWD and PDFF, and ATI and PDFF measurements (SPSS, Version 24, IBM, Boston, MA, USA). The correlations were classified as slight (from 0.00 to 0.25), moderate (from 0.25 to 0.50), good (from 0.50 to 0.75) and very good (from 0.75 to 1.00) [26]. A *p*-value < 0.05 was considered statistically significant (with a 95% confidence interval). In addition, a descriptive statistical analysis was carried out.

## 3. Results

We performed a retrospective analysis of the US and MRI data of 11 male and 4 female patients who underwent liver sonography and MRI for hepatic steatosis between November and December 2020. The patients were aged between 34 and 80 (mean 59.7 ± 14.9). The etiology of fatty liver disease was non-alcoholic fatty liver disease (NAFLD) in three cases and chemotherapy due to hepatic metastases from a colorectal cancer in one case. The other patients (n = 11) had histories of present or past illnesses from liver cirrhosis (n = 3), hepatitis B (n = 1) or liver tumor therapy (n = 7). Applying a reported cut-off value of 5%, PDFF sequences from MRI examinations proved hepatic steatosis in 4 out of 15 cases, with the fat-signal-fraction rates ranging from 0.7 to 14% (mean 3.84% ± 3.6). Three patients presented measurements suggesting mild hepatic steatosis, and one patient presented measurements suggesting moderate hepatic steatosis.

The SWS measurements in patients with fatty liver ranged from 1.24 to 4.43 m/s (mean 2.8 m/s ± 0.76), and the stiffness values ranged from 4.9 to 62.5 kPa (mean 20.8 kPa ± 16.8). Cut-off values of 2.5 m/s and 20.4 kPa were calculated using a ROC analysis and the Youden index. By applying these cut-off values, three out of four patients with and eight out of eleven patients without MRI-proven hepatic steatosis could be correctly identified. Color-coded SWE charts suggested significant or severe steatosis in nine cases, of which three patients had corresponding PDFF results; moderate steatosis in one patient with MRI-proven steatosis; and no fatty liver in four patients. In accordance with the PDFF results, the SWE’s sensitivity and specificity for steatosis detection were 75% and 72.7%, respectively, with *p* = 0.001 for elasticity measurements and *p* > 0.05 for the SWS measurements. The AUC for the SWE was 0.73 (0.46–0.99, *p* > 0.05). A moderate correlation (r = 0.27, *p* > 0.05) with the PDFF’s fat-signal-fraction measurements was observed.

The qualitative SWD analysis showed five cases with orange-, eight cases with red- or yellow-, and two cases with green-colored charts, suggesting thirteen cases of steatosis. The SWD measurements ranged from 10.5 to 23.1 m/s/kHz (mean 16.5 m/s/kHz ± 4.58) and showed significant correlations (r = 0.55, *p* = 0.034) with the PDFF’s steatosis detection. The AUC was 0.73 (0.35–1). A calculated SWD cut-off of 18.5 m/s/kHz as well as color-coded charts could correctly identify all four patients with liver steatosis. Out of eleven patients without fatty livers, ten were correctly identified via quantitative analysis and two via qualitative analysis. The sensitivity and specificity of the SWD measurements were 75% and 90.9%, respectively (*p* < 0.05).

The ATI measurements ranged from 0.5 to 0.8 dB/cm/MHz (mean 0.6 dB/cm/MHz ± 0.12). One patient with and one without hepatic steatosis showed corresponding color-chart changes suggesting moderate ATI elevation. Nine patients showed green-coded charts, suggesting no steatosis in the qualitative analysis. A cut-off value of 0.59 dB/cm/MHz was calculated to differentiate between patients with hepatic steatosis and those with no signs of fatty livers. Therefore, two out of four patients with steatosis and six out of eleven patients without fatty livers were correctly identified, resulting in a sensitivity rate of 50% and a specificity rate of 54.5%. The other two patients with PDFF-proven steatosis showed ATI values close to the calculated cut-off (0.55 and 0.56 dB/cm/MHz). The ATI measurements showed good correlations with the PDFF results (r = 0.58, *p* = 0.024). An AUROC of 0.534 (0.18–0.88) was calculated for the ATI (*p* < 0.05).

**Figure 5:**
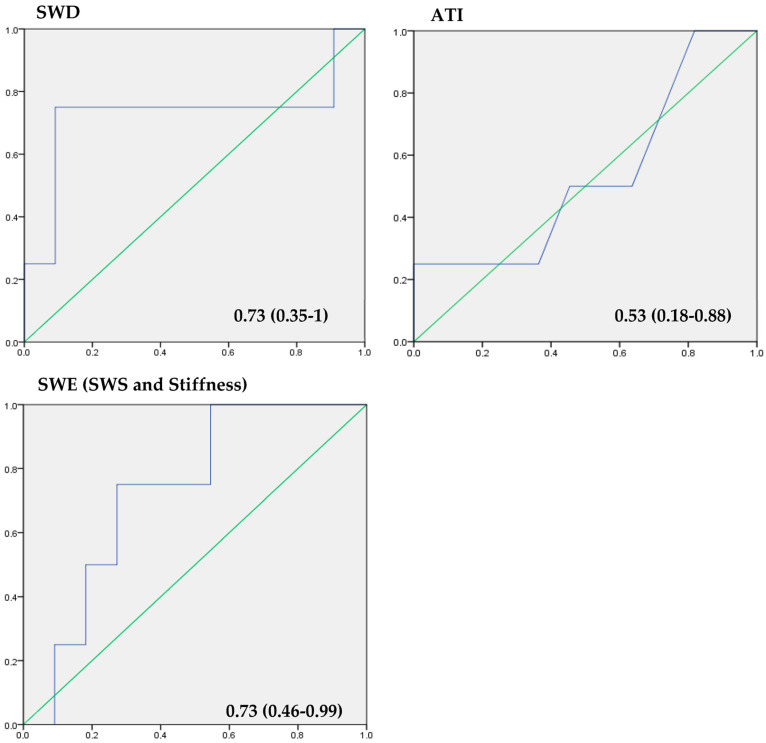
AUROC analysis of the applied elastography techniques: SWE (incl. shear-wave speed and stiffness measurements), SWD and ATI. The *x*-axis represents the reciprocal specificity and the *y*-axis represents sensitivity.

An overview of the results is provided in Table 1. The AUROC values for the SWE, SWD and ATI results are summarized in Figure 5.

## 4. Discussion

This study compared different US elastography techniques using qualitative parameters and the quantitative measurements of SWS, stiffness, SWD and ATI. They were compared to established MRI PDFF mappings of the liver’s fat-signal fraction for the detection of hepatic steatosis, applying specific cut-off values as a novelty. Although only preliminary findings were reported, the results suggested that SWD and ATI correlated better than the SWE to the PDFF mapping measurements, which has been established as the non-invasive imaging gold standard for fatty liver disease [19,24]. The proposed cut-off values for all the analyzed techniques represent initial suggestions which could be used to differentiate patients with hepatic steatosis.

Modern US elastography techniques, including SWE, SWD and ATI, provide the advantage of being cost-effective, mobile and quickly applicable screening methods with high diagnostic accuracy in the staging of various liver diseases [7,13,15,23,27]. Hepatitis, steatosis and iron storage as well as fibrosis and cirrhosis can be assessed non-invasively using SWE, which shows good correlations with the MRI and liver biopsy results, which are considered to be the gold standard in the diagnosis of hepatic diseases [16,19,24,28]. Not all patients are able to undergo MRI due to claustrophobia, renal insufficiency or the presence of implanted, non-compatible devices; therefore, a transabdominal US can be used as a suitable alternative in such cases. Furthermore, MRI is a more cost- and time-consuming imaging modality, which might make it less suitable for use in screening purposes [19].

Before the implementation of modern elastography techniques, B-mode US was used to compare differences in the signal attenuation of liver and renal parenchyma in order to diagnose hepatic steatosis. Due to its nature, this method of steatosis diagnostics is very subjective and might not be reproducible, which makes quantifiable measurements preferable [8,11].

The high value of ATI measurements in the identification and staging of patients with hepatic steatosis has already been reported by a few authors. They compared ATI values with biopsy results, TE measurements and MRI PDFF results and, where applicable, they provided similar cut-off values of 0.59–0.69 dB/cm/MHz as reported in this study (0.59 dB/cm/MHz) [12,13,15,16,19,20,25,29]. The consistency of the measurements across different publications might be explained by the fact that the degree of steatosis is the only factor affecting the acoustic coefficient resulting from the dispersion slope, whereas liver fibrosis, cirrhosis or inflammatory diseases do not have any impact [16]. Our findings suggested slight differences compared with other findings in the recent literature. Indeed, as sensitivity in this preliminary study was relatively low, the qualitative analysis of color-coded charts was not specific and the correlation with the PDFF results was moderate, which might have been due to the small population size [12,21,30]. Nevertheless, the fact that the other two patients with steatosis in this study had ATI values very close to the calculated cut-off value—thus resulting in relatively higher sensitivity—suggests that ATI is a valuable tool for comparable measurements in many stages of the diagnostics and therapy of fatty liver diseases of different etiologies.

In this study, shear-wave dispersion proved to be the most effective technique in the detection of hepatic steatosis compared with ATI and SWE, providing the highest sensitivity and specificity rates. This might be explained by the influence of viscoelasticity changes on SWD measurements, which are present in fatty liver disease [17,18,23]. The elevated viscoelasticity of hepatocytes caused by lipid accumulation inside the cells and consecutive necroinflammation is displayed and quantifiable in SWD imaging [23,31]. However, the accompanying elasticity changes (which can result from simultaneous fibrosis, cirrhosis or severe inflammation) can affect SWD measurements. Therefore, other studies in the recent literature reported the application of SWD measurements for the detection and staging of inflammatory hepatic diseases and preoperative or post-liver transplantation fibrotic diseases, but not for the assessment of hepatic steatosis as this study’s findings suggest [5,15,17,23,31,32]. In this study, SWD measurements showed good correlations with MRI PDFF measurements compared with ATI values (r = 0.55 vs. r = 0.58), suggesting SWD’s high diagnostic performance in the assessment of steatosis.

However, the sensitivity and specificity rates were higher when SWD and SWE were used for steatosis detection compared with ATI, although ATI showed the highest correlation with the PDFF findings.

Regarding the application of SWE for the diagnosis of liver diseases, numerous studies reported the efficiency of elastography in the diagnosis and staging of liver fibrosis/cirrhosis and inflammatory diseases using various shear-wave techniques, i.e., pSWE, 2D SWE or TE [6,9,10,14,33]. However, the international EFSUMB’s guidelines only recommend SWE measurements for the diagnosis and prediction of complications in chronic hepatitis B and C, and not for NAFLD or ALD, which are the main causes of hepatic steatosis in the early stages of the diseases [1,2]. Our results confirm the impressions of other authors who suggested a high feasibility for the assessment of fatty liver disease based on SWE [5,6,14]. Applying cut-off values of 2.5 m/s and 20.4 kPa, sensitivity and specificity rates of >73% could be obtained in this preliminary and relatively small study population. This suggests that the accuracy rates might rise with more patients, which we are currently examining in an ongoing prospective study. Although SWE seemed to have a good feasibility for detecting steatosis, the correlation with the PDFF results was moderate only. This could also be due to the fact that SWE measurements are limited in mild-steatosis assessments as reported in some studies [34,35]. Shear-wave measurements are primarily affected by elasticity changes in the liver tissue, which is not an early result of lipid accumulation in hepatocytes in early-steatosis stages. The soft-lipid sediments change the viscosity in the early stages first, which can better be displayed and quantified using ATI or SWD, before a continuous fibrotic remodeling of the liver tissue occurs in advanced stages of liver steatosis, which enables a more accurate detection of the disease by SWE. These results were confirmed by other authors, who recommend the application of SWE for fibrosis/cirrhosis or hepatitis assessments, reporting sensitivity rates of up to 96% in the diagnosis and staging of liver fibrosis and chronic hepatitis B/C infections [4,5,6,7,9,10,13].

A limitation of this study was the relatively small study population, which is explained by the fact that we wanted to publish preliminary results as a proof of principle. Therefore, we examined consecutive patients in a four-week period only using USs and MRIs, which was limited by the modalities’ capacities. After the application of our inclusion criteria, the groups of patients with (n = 4) and without (n = 11) liver steatosis were unequal, which we could not change at the time. Nevertheless, in each case, five measurements were acquired using all the techniques examined, and a sufficient database was thus created. Furthermore, we focused on the diagnosis of hepatic steatosis and did not take into account the influence of synchronic inflammatory, fibrotic or cirrhotic changes. Further limitations include the monocentric, retrospective study design and the use of a single supplier’s US machine for the elastography measurements. Larger studies with prospective study designs and large numbers of participants are therefore warranted.

## 5. Conclusions

The results of this study suggest that SWD and SWE are the most sensitive, non-invasive tools for the detection and staging of hepatic steatosis, with good correlations with MRI PDFF mapping as the gold standard. Nevertheless, ATI showed the highest correlation with the PDFF results. The combined use of all techniques is a promising way to confirm or rule out the presence of different stages of hepatic steatosis.

## Figures and Tables

**Table 1:** Overview of the quantitative analysis results of SWE, SWD and ATI and the corresponding statistical analysis results of ROC/AUROC analysis, Pearson’s coefficient and *t*-test application for paired samples. A *p*-value < 0.05 was considered statistically significant (*). Sensitivity and specificity rates are based on the calculated cut-off value.

	Mean ± SD (Range)	AUC (95% CI)	Sensitivity	Specificity	r	*p*-Value	Cut-Off Value
SWE (m/s)	2.8 ± 0.76 (1.24–4.43)	0.73 (0.46–0.99)	75%	72.7%	0.27	>0.05	2.5
SWE (kPa)	20.8 ± 16.8. (4.9–62.5)	0.73 (0.46–0.99)	75%	72.7%	0.27	0.001 *	20.4
SWD (m/s/kHz)	16.5 ± 4.58 (10.5–23.1)	0.73 (0.35–1)	75%	90.9%	0.55	0.034 *	18.5
ATI (dB/cm/MHz)	0.6 ± 0.12 (0.5–0.8)	0.53 (0.18 –0.88)	50%	54.5%	0.58	0.024 *	0.59

## Data Availability

The data presented in this study are available on request from the corresponding author. The data are not publicly available due to ongoing research. The findings presented in this study are preliminary.
